# Varicella vaccine dose depended effectiveness and waning among preschool children in Hong Kong

**DOI:** 10.1080/21645515.2019.1663121

**Published:** 2019-10-23

**Authors:** Yung-Wai Desmond Chan, W John Edmunds, Hong-Lam Chan, Miu-Ling Wong, Ka-Wing Albert Au, Shuk-Kwan Chuang, Albert Jan van Hoek, Stefan Flasche

**Affiliations:** a Communicable Disease Division, Surveillance and Epidemiology Branch, Centre for Health Protection, Department of Health Hong Kong SAR Government, Kowloon, Hong Kong SAR; b Department of Infectious Disease Epidemiology, Faculty of Epidemiology and Population Health, London School of Hygiene and Tropical Medicine, London, UK

**Keywords:** Chickenpox, chickenpox vaccine, immunity, vaccine effectiveness

## Abstract

In Hong Kong, universal varicella vaccination was introduced in July 2014 with a two-dose schedule but the vaccines had been available in the private market since 1996. With data from varicella notification and surveys on immunization coverage, we used the screening method to estimate dose-specific varicella vaccine effectiveness (VE) among preschool children in Hong Kong before universal vaccination. We estimated the VE of one- and two-dose varicella vaccination against all notified varicella as 69.4% (95% confidence interval (95% CI) 69.5–71.2) and 93.4% (95% CI 91.7–94.7), respectively. We found that VE did not decrease with time since receipt. Varicella vaccine was more effective against complications (85.4% [95% CI 48.8–95.8] for one dose and 100% [95% CI –Inf to 100] for two doses) and against hospital admission (75.2% [95% CI 53.4–86.8] for one dose and 93.1% [95% CI 47.1–99.1] for two doses). Lower protection of one-dose varicella vaccine resulted in breakthrough varicella. Under universal vaccination, second-dose varicella vaccine (given as combined measles, mumps, rubella and varicella vaccine) was first scheduled for children when they reach primary one (about 6 years of age) and was recently advanced to 18 months of age. Shortening the interval between the first dose and second dose of varicella vaccination should reduce breakthrough varicella and outbreaks in preschool.

## Introduction

Universal varicella vaccination (UVV) in Hong Kong was introduced in July 2014 for children born in 2013 and after with a two-dose schedule at 12 months and around 6 years of age (at the start of primary school).^1^ Monovalent varicella vaccine (mVV) is provided as the first dose whilst combined measles, mumps, rubella, and varicella (MMRV) is provided as the second dose. However, before the start of UVV, varicella vaccine was licensed for private market use in 1996 with no clear schedule and the first dose vaccination uptake gradually reached up to 50% among preschool children.^2^ The private market was dominated by mVV from GSK, Sanofi, and MSD (Table 1). All three products consisted of live attenuated varicella virus (Oka strain) with at least 1350 plaque-forming unit.^3^ We found that most vaccinated preschool children only received one dose of vaccine, with median age of vaccination ranging from about 15 to 20 months of age.^2^ We previously reported the burden of varicella shifting to older children during the period of increasing vaccination uptake in the private market. However, the extent to which this increase in varicella vaccination contributed to the change in epidemiology remains uncertain.^2^

**Table 1.** Type of varicella vaccine received for vaccinated survey participants, 2009 and 2012 immunization survey for preschool children, Hong Kong.

		First dose				Second dose			
		2009		2012		2009		2012	
Type of varicella vaccine		*N*	%	*N*	%	*N*	%	*N*	%
mVV	Varilix (GSK)	496	32.7	658	39.7	12	54.5	13	19.7
	Okavax (Sanofi)	195	12.8	171	10.3	0	0.0	8	12.1
	Varivax (MSD)	68	4.5	118	7.1	3	13.6	5	7.6
	Manufacturers from mainland China	14	0.9	13	0.8	0	0.0	0	0.0
	GreenCross Korea	0	0.0	1	0.1	0	0.0	0	0.0
MMRV	PriorixTetra (GSK)	0	0.0	0	0.0	0	0.0	4	6.1
	Exact brand and manufacturer unclear	0	0.0	0	0.0	0	0.0	3	4.5
Unknown		746	49.1	695	42.0	7	31.8	33	50.0
Total		1519	100.0	1656	100.0	22	100.0	66	100.0

mVV: monovalent varicella vaccine

MMRV: combined measles, mumps, rubella﻿ and varicella vaccine

Vaccinees developing modified (less severe) varicella is often referred to as breakthrough infections.^3^ In the United States, the frequent reporting of breakthrough infections in one-dose recipients with low protection against varicella^4,5^ has led to the implementation of two-dose program, with the first dose scheduled at 12–15 months while the second dose was scheduled at 4–6 years. Yet, in Hong Kong, the second dose was first scheduled at about 6 years of age and hence at least 5 years following the first dose of varicella vaccine. The decision to space out the two doses was largely on programmatic grounds and this schedule may result in a high number of breakthrough cases before receipt of a second dose because of limited effectiveness^6^ or potential waning following a single dose of varicella vaccine.

We used the screening method to estimate the direct effect of varicella vaccine in Hong Kong before UVV. The primary objective of this study is to estimate the effectiveness of varicella vaccine against varicella infections of all severity in preschool children in Hong Kong. Secondary objectives include estimating varicella vaccine dose-specific effectiveness, whether vaccine effectiveness (VE) waned with time and VE against complications and severe diseases.

## Patients and methods

We used the screening method^7,8^ to estimate the effectiveness of varicella vaccine among children aged 3–5 years in Hong Kong. In contrast to vaccine efficacy, which is typically defined as the direct effect of a vaccine measured in pre-licensure clinical trials, VE commonly referred to the estimated protection attributed to a vaccine under post-licensure, field conditions.^9^ Post-licensure VE is commonly estimated using case–control and cohort studies by comparing the attack rate or incidence rate among vaccinated and unvaccinated cases. On the other hand, the screening method is used to estimate VE by making use of surveillance data consisting of the vaccination coverage among the cases and the source population.^7,8^ In our study, we compared the proportion of childhood varicella cases who were vaccinated (PCV) with the proportion of the children vaccinated in the source population (PPV) for the corresponding age cohort to obtain the VE.

### Data sources

PPV was obtained from territory-wide immunization surveys on preschool children conducted by the Department of Health (DH). The surveys were cross-sectional studies using stratified cluster sampling of preschools in Hong Kong to estimate vaccine uptake, which was the proportion of children receiving a particular vaccine. Vaccination status was ascertained by reviewing documented vaccination records. As the screening method is disproportionately prone to error when the PPV and PCV is very high or very low,^7^ we chose surveys conducted in 2009, 2012, and 2015 covering cohorts with an intermediate uptake of 23.8–51.0% in the private market. This provided us with additional confidence for a robust VE estimation before UVV as first dose uptake for children eligible for universal vaccination has reached 99%.^10^ About 5% of preschools were selected in these three surveys, with 6051–8522 children aged 2–5 from sampled preschools were recruited in these three surveys. We used varicella vaccine uptake of children aged 3–5 only as preschool attendance rate for children of these ages were high,^11^ compared to the 50% attendance rate for those aged 2 years. Details of the surveys have been reported elsewhere.^2^ Survey data readily available for analysis included demographics (including birth year, sex, place of birth, and residence) and varicella vaccination history (including vaccination status, number of dose(s) received, and date of vaccination (for 2015)). Two independent reviewers retrospectively digitalized the number of dose(s), date of vaccination, and vaccine product for the 2009 and 2012 surveys from stored paper forms. We described the varicella vaccination uptake for different birth cohorts, including the number and proportion of surveyed children that received varicella vaccine, as well as the timeliness of vaccination, as indicated by median and interquartile range of the age of vaccination.

Varicella is a notifiable disease in Hong Kong. The DH receives varicella notifications from doctors in public and private sectors, as well as from varicella outbreaks from schools and institutions. Probable cases were defined as those with the clinical presentation of acute onset of diffuse (generalized) papulovesicular rash without other apparent cause or atypical (milder) clinical presentation for those with varicella vaccination history. Confirmed cases were defined as probable cases with either laboratory confirmation or epidemiologically linked to a confirmed case. We included all confirmed and probable cases in our analyses. Parents and/or doctors were interviewed using a standard questionnaire to obtain the demographics (including age, gender, place of residence, and travel history during the incubation period), clinical information (including date of onset, any varicella-related complications, hospitalization, and fatality), and varicella vaccination history (vaccination status, number of dose(s) received, and age of last varicella vaccination). Number of dose(s) was recorded for cases reported in 2012 onwards. However, since second-dose varicella vaccination uptake for children surveyed in 2009 was only 0.5% or below (Table 2) and the information on number of dose(s) was not collected for varicella cases reported in 2009, we assumed that all vaccinated varicella cases in 2009 received only one dose. Doctors and/or hospitals were contacted to obtain clinical information when parents reported that their children developing varicella-related complications and/or were admitted to hospitals. We extracted information from the questionnaire into an electronic database. To match with the children captured by the immunization surveys (source population), we selected reported cases aged 3–5 years attending preschools in 2009, 2012, and 2015. We excluded cases who did not attend preschools, who did not reside in Hong Kong or imported cases (i.e. those who had a travel history during the incubation period), or who resided in residential homes from our analyses as these children were unlikely to be sampled in the immunization surveys. Children fulfilling one of the following criteria were regarded as attending preschools: attendance being confirmed by parents, notifications by preschools, or cases identified during investigation of varicella outbreaks in preschools. We described the demographics of varicella cases included in our analysis.

**Table 2.** Varicella vaccination uptake and age of vaccination in preschool children surveyed and preschool children with varicella reported in Hong Kong, 2009, 2012, and 2015.

Varicella vaccination among preschool children surveyed							
			At least 1 dose	1st dose		2nd dose	
Survey year	Age (years)/cohort	Number surveyed	Number received (%)	Number received (%)	Median age (months) at 1st dose (IQR)	Number received (%)	Median age (months) at 2nd dose (IQR)
2009	5/2003	1666	397 (23.8)	375 (22.5)	25.5 (16.7–34.5)	7 (0.4)	40.0 (38.7–64.1)
	4/2004	1726	509 (29.5)	405 (23.5)	24.9 (16.5–32.4)	9 (0.5)	40.7 (35.9–49.7)
	3/2005	1819	613 (33.7)	526 (28.9)	23.0 (15.6–28.8)	6 (0.3)	37.0 (35.2–37.4)
2012	5/2006	1596	430 (26.9)	375 (23.5)	20.6 (14.7–29.5)	24 (1.5)	62.0 (39.3–65.5)
	4/2007	1834	614 (33.5)	546 (29.8)	19.0 (14.2–27.9)	22 (1.2)	48.7 (36.6–51.7)
	3/2008	1815	612 (33.7)	575 (31.7)	18.4 (14.1–25.9)	20 (1.1)	35.4 (27.0–40.9)
2015	5/2009	2276	1034 (45.4)	924 (40.6)	19.2 (14.3–27.4)	110 (4.8)	51.0 (39.9–59.2)
	4/2010	2488	1216 (48.9)	1046 (42.0)	16.3 (14.0–23.5)	170 (6.8)	34.9 (24.6–50.4)
	3/2011	2831	1443 (51.0)	1172 (41.4)	15.6 (13.5–21.9)	271 (9.6)	22.6 (17.2–30.4)
Varicella vaccination among cases reported to the DH							
			At least 1 dose	1st dose		2nd dose	
Notification year	Age (years)	Number reported	Number received (%)	Number received (%)	Median age (year) at 1st dose (IQR)	Number received (%)	Median age (month) at 2nd dose (IQR)
2009^4^	5	684	130 (19.0)	130 (19.0)	2.0 (1.0–3.0)	–	–
	4	751	121 (16.1)	121 (16.1)	2.0 (1.0–2.0)	–	–
	3	653	95 (14.5)	95 (14.5)	1.0 (1.0–2.0)	–	–
2012	5	861	177 (20.6)	147 (17.1)	1.0 (1.0–2.0)	6 (0.7)	3.0 (2.5–3.0)
	4	951	182 (19.1)	146 (15.4)	2.0 (1.0–2.0)	7 (0.7)	3.0 (2.0–3.0)
	3	727	126 (17.3)	105 (14.4)	1.0 (1.0–2.0)	3 (0.4)	1.0 (1.0–1.5)
2015	5	838	171 (20.4)	136 (16.2)	1.0 (1.0–2.0)	15 (1.8)	2.0 (2.0–5.0)
	4	866	189 (21.8)	143 (16.5)	1.0 (1.0–2.0)	20 (2.3)	2.0 (1.0–2.0)
	3	675	110 (16.3)	89 (13.2)	1.0 (1.0–1.3)	11 (1.6)	2.0 (1.0–2.0)

Note:

(1) Survey respondents and reported cases aged three to five were included in this study.

(2) Survey respondents and reported cases with missing vaccination information were not included in the above table. Those who were vaccinated with unknown dose was not shown.

(3) Number and the proportion (%) of children received varicella vaccine for different doses was presented in the above table. Proportion was computed by the number of children received the vaccine divided by the number surveyed/ reported in each stratum.

(4) Number of varicella vaccine received was not collected for cases reported in 2009. In view of low second-dose uptake for children surveyed in 2009, all vaccinated varicella cases reported in 2009 were assumed to have received only one dose.

(5) Age of vaccination for survey respondents was computed by subtracting date of vaccination with date of birth. Median and interquartile range (IQR) of the age of vaccination was presented in the above table to indicate timeliness of vaccination.

(6) Age of vaccination for varicella cases was collected by a standardized questionnaire for which the precision is up to year only.

### Assessing waning immunity

To assess whether the effectiveness of varicella vaccine waned with time, time since last vaccination was computed for vaccinated cases by subtracting the age at most recent varicella vaccination by the age at disease onset. Waning refers to the loss of vaccine protection with time. Similar computation is needed for unvaccinated cases when VE is estimated by the screening method.^12,13^ Studies carried out when a common vaccination schedule was in place could impute the time of vaccination by assuming that unvaccinated cases would have received the vaccine under the recommended schedule.^12,13^ In our study, varicella VE was estimated before universal vaccination and the age of vaccination through private market in the source population was highly diverse (Table 2). Therefore, the time since last (hypothetical) vaccination for unvaccinated cases cannot be approximated by simply using the recommended age of vaccination or the median age of vaccination in the healthy population. Assuming the time of vaccination for unvaccinated cases would have been comparable to those in the population should they choose to vaccinate, we imputed the age of (hypothetical) vaccination for unvaccinated cases from the observed vaccination timing in the population, i.e. survey respondents vaccinated against varicella (Supplementary Figure 1). In order to obtain a dataset with the age of (hypothetical) vaccination for unvaccinated cases completely imputed, we first imputed missing values of vaccination-related variables among vaccination survey respondents and varicella cases to obtain complete datasets. The imputation process is described in detail in Supplementary Figure 1. A maximum number of 25 iterations for each imputation was chosen as 20–30 iterations were suggested to be sufficient in achieving convergence of variables under imputation, which is fewer than other Gibbs sampling methods.^14^ We created 500 multiply imputed datasets.

### Estimation of VE

Following Orenstein,^7^ we estimated VE via:

$$VE=1-\left(PCV\right)\left(1-PPV\right)/\left[\left(1-PCV\right)\left(PPV\right)\right]$$

We computed PPV specific to different years (2009, 2012, and 2015), age (3, 4, and 5 years), and dose (at least one, one and two doses). PPV were then matched to each varicella cases based on their year of survey/notification, age, and number of dose(s) received. We computed the VE following the approach suggested by Farrington,^15^ which was also adopted and described in detail in other VE studies:^12,16,17^

$$In\left[P/\left(1-P\right)\right]=c+In\left[PPV/\left(1-PPV\right)\right]$$

where *P* is the probability of vaccination status of reported cases fitted as a binary outcome (vaccinated or not), with log odds of the matched PPV (In[PPV/(1-PPV)]) as an offset in a logistic regression model. VE was obtained as VE = 1 – exp(*c*) where c was the estimated constant in the regression model.

VE estimates were obtained for each imputed dataset and then pooled to give a single estimate for which the within- and between-imputation variance was accounted for according to Rubin’s rules.^14^ We reported the pooled VE estimates alongside their 95% confidence intervals in our results.

We computed the varicella VE of different doses (any, one and two doses (for 2012 and 2015)) against different outcomes including all varicella infections, varicella with complications, and varicella-related hospitalizations. In addition, we included time since vaccination as a covariate in the logistic regression model for VE against all varicella infections and obtained the VE at different time periods since vaccination (0, 1, 2, 3, and 4 years) to assess whether there was evidence of waning immunity.

### Software

All analyses were done in R^18^ including the multiple imputation via the MICE package.^14^

## Ethical approval

Ethical approval was obtained from the Observational/Interventions Research Ethics Committee of the London School of Hygiene & Tropical Medicine as part of a modeling study of varicella vaccination in Hong Kong (LSHTM ethics ref: 11852).

## Results

### Varicella vaccination uptake in the community

Uptake for at least one dose of varicella vaccine gradually increased from about 25% for preschool children surveyed in 2009 to about 50% for those surveyed in 2015 (Table 2). Most preschool children only received one dose of varicella vaccine. Second-dose vaccine uptake was about 1% or less in 2009 and 2012, and it increased to 5–10% in 2015. Age at vaccination varied greatly among different cohorts, especially for the second dose (Table 2).

### Varicella notifications

A total of 7302 varicella cases aged 3–5 years were recorded in 2009, 2012, and 2015, corresponding to a notification rate of 1534 per 100,000. After excluding cases that were imported (i.e. those who had a travel history during the incubation period) (33), lived in residential care homes (4), and did not attend preschools (259), we included 7006 cases in our analyses. Only two cases were immunosuppressed (one case of nephritis and one case of pre-B acute lymphoblastic leukemia). Twenty-nine cases (0.4%) had complications, among which 18 were scarlet fever, one case each of pneumonia, febrile convulsion, cellulitis and abscess due to methicillin-resistant *Staphylococcus aureus*, and mild skin infection. Exact complication type was not known for the remaining seven cases. Seventy-five cases (1.1%) were admitted to hospitals but no fatal case was identified.

The vaccination uptake for varicella cases slightly increased from 16.6% in 2009 to 19.1% in 2012 and 19.8% in 2015 (Table 3). For cases reported in 2012 and 2015, less than 3% had received a second dose (Table 2). The proportion of hospital admissions was comparable for children of different vaccination status albeit sample size was small (1.1% (95% CI 0.9–1.4) among unvaccinated, compared to 1.0% (95% CI 0.6–1.8) and 1.6% (95% CI 0.3–8.6) among one- and two-dose recipients). Among two-dose recipients, there was no case with complication and only one case was admitted to hospital.

**Table 3.** Characteristics of varicella notifications aged 3–5 years^1^, Hong Kong, 2009, 2012, and 2015.

		2009 (*n* = 2088)		2012 (*n* = 2539)		2015 (*n* = 2379)		Total (*n* = 7006)	
Characteristics		*N*	%	*N*	%	*N*	%	*N*	%
Female gender		923	44.2	1156	45.5	1063	44.7	3142	44.8
Age (years)	3	653	31.3	727	28.6	675	28.4	2055	29.3
	4	751	36.0	951	37.5	866	36.4	2568	36.7
	5	684	32.8	861	33.9	838	35.2	2383	34.0
Clinical condition	Immunosuppression	0	0.0	1	0.0	1	0.0	2	0.0
	Complications	3	0.1	13	0.5	13	0.5	29	0.4
	Hospitalization	19	0.9	26	1.0	30	1.3	75	1.1
Varicella vaccination		346	16.6	485	19.1	470	19.8	1301	18.6
No. of dose received^2^	1	NA	NA	398	82.1	368	78.3	766	58.9
	2	NA	NA	16	3.3	46	9.8	62	4.8
	Unknown	346	100.0	71	14.6	56	11.9	473	36.4
Age of last vaccination (year)^2^	<1	1	0.3	2	0.4	2	0.4	5	0.4
	1	122	35.3	215	44.3	234	49.8	571	43.9
	2	142	41.0	142	29.3	123	26.2	407	31.3
	3	40	11.6	55	11.3	37	7.9	132	10.1
	4	16	4.6	15	3.1	10	2.1	41	3.2
	5	2	0.6	2	0.4	7	1.5	11	0.8
	Unknown	23	6.6	54	11.1	57	12.1	134	10.3

Note:

^1^Two hundred and ninety-six cases were excluded from the analyses and thus not included in the above table (imported: 33, residential care homes: 4, did not attend preschool: 259).

^2^Among those vaccinated with varicella vaccines.

### Varicella VE

We estimated the VE of one-dose varicella vaccination against all notified varicella as 69.4% (95%CI 67.5%–71.2). The respective two-dose VE was substantially higher at 93.4% (95%CI 91.7–94.7). We did not find evidence for waning immunity of varicella vaccination against all notified varicella. For one-dose recipients, the estimated VE did not decrease significantly with time since receipt (Table 4). On the other hand, we found that two-dose VE increased with time since receipt. Varicella vaccine was more effective against complications: 85.4% (95%CI 48.8–95.8) for one dose and 100% (95%CI –Inf to 100) for two doses. The effectiveness of varicella vaccines against hospital admission was 75.2% (95% CI 53.4–86.8) and 93.1% (95% CI 47.1–99.1) for one- and two-dose recipients, respectively (Table 4).

**Table 4.** Vaccine effectiveness for different doses of varicella vaccine against all varicella, varicella with complications, and varicella admissions among preschool children aged 3–5 years in Hong Kong.

Outcome/dose		Vaccine effectiveness % (95% CI)
All varicella		
Any dose		68.7 (66.8–70.5)
Time since vaccination (year)	0	68.7 (63.4–73.3)
	1	68.7 (61.1–74.8)
	2	68.7 (58.7–76.3)
	3	68.7 (56.1–77.7)
	4	68.7 (53.3–79.0)
1 dose		69.4 (67.5–71.2)
Time since vaccination (year)	0	70.8 (65.7–75.2)
	1	70.2 (62.8–76.2)
	2	69.7 (59.8–77.3)
	3	69.2 (56.4–78.3)
	4	68.7 (52.8–79.2)
2 doses		93.4 (91.7–94.7)
Time since vaccination (year)	0	86.4 (77.2–92.0)
	1	90.6 (80.0–95.6)
	2	93.5 (82.5–97.6)
	3	95.5 (84.7–98.7)
	4	96.9 (86.6–99.3)
Complication		
Any dose		86.0 (50.9–96.0)
1 dose		85.4 (48.8–95.8)
2 doses		100.0 (–Inf to 100.0)
Hospital admission		
Any dose		74.2 (52.6–86.0)
1 dose		75.2 (53.4–86.8)
2 doses		93.1 (47.1–99.1)

Note:

• Time since vaccination was included as a covariate only in the logistic regression model for VE against all varicella infections.

• Since dose of vaccine received was not collected for varicella cases reported in 2009 and the second-dose varicella vaccination uptake in the population is very low, all vaccinated cases reported in 2009 were assumed to have only received one dose of vaccine. As such, VE estimation for any dose (ever vaccinated) and ﻿one dose was based on all 3 study years (2009, 2012, and 2015) whereas two doses was based on data from 2012 and 2015 only.

• Variable “dose no.” was not added in the regression model when estimating VE for any dose due to issue in model convergence. As such, VE for any dose was not adjusted for no. of doses received.

## Discussion

We used the screening method to estimate the varicella VE among preschool children in Hong Kong. We showed that one-dose varicella vaccination conferred moderate direct protection [69.4% (95% CI 67.5–71.2)] against notified varicella whilst two doses conferred strong direct protection [93.4% (95% CI 91.7–94.7)]. VE against complications and hospital admissions was also generally higher for those who received two doses, though numbers were too small to conclude of superiority. We did not find any evidence to support concerns that vaccine protection from one dose would wane before children entering primary school.

Our VE estimates are largely comparable to a recent meta-analysis of post-licensure VE studies^6^ which also showed that one-dose varicella vaccine (mostly mVV) is moderately effective for preventing disease of any severity [81% (95% CI 78–84%)] but two-dose varicella vaccine is highly effective at 98% (95% CI 97–99%). We also found that the effect of varicella vaccination persisted in the first few years after vaccination with no apparent decline in VE. For two-dose vaccinees, there was a general trend of increasing effectiveness with time since vaccine receipt. There are a few possible explanations behind this observation. First, as children aged, they had more exposure to circulating wild-type varicella, which might boost up their immunity. Second, vaccinees who failed to develop adequate immunity would be infected and became immune to further varicella infections. Thus, the number of vaccinated yet susceptible children would decrease with time and contribute less to the varicella reported in later years. This would result in lower PCV and higher VE in later years. Third, there were only 62 reported cases having received two doses of varicella vaccine and the estimation of two-dose VE by year since vaccination was more prone to error due to small sample size. Clinical trials of Varivax showed that vaccinees who were initially protected lost their protection rather quickly,^19^ while some studies showed that antibodies against varicella were persistent among vaccinees after 10- to 20-year follow-up, though boosting by circulating wild-type VZV cannot be ruled out.^20^ Systematic review found that waning immunity for single-dose vaccination was not conclusive,^20,21^ and long-term protection of up to 14 years had been demonstrated for two-dose regimen in the World Health Organization’s systematic review.^22^

As one-dose varicella vaccine induces only moderate protection, breakthrough infections are often observed in one-dose vaccinees.^4,5^ Although waning immunity after first dose varicella vaccination remains inconclusive, the lower VE is generally believed to be a result of primary vaccine failure which mainly occurred after first dose, as evidenced by only 76–84% of one-dose recipients seroconverted (assessed by fluorescent antibody to membrane antigen test, the most specific laboratory test for serological correlates of varicella infection and vaccination).^21^ Furthermore, IgG geometric mean concentration of subjects receiving the first dose MMRV of MSD (ProQuad) was lowest for varicella and the boosting effect after the second dose was much more profound for varicella,^3^ suggesting that immune response following only one dose against varicella is likely incomplete. In addition to nonconverter (primary vaccine failure), mild breakthrough infections occurring among seroconverted vaccinees (commonly defined as those who had >5 glycoprotein enzyme-linked immunosorbent assay units/mL) may be a result of partial or “leaky” protection.^4,21^ Thus, second-dose varicella vaccination is important in completing the incomplete immune response following first-dose vaccination and maximizing the impact of varicella vaccination. After the start of a one-dose program in 1995, the United States switched to a two-dose regimen in 2006 as varicella outbreaks persisted even among the highly vaccinated parts of the population.^23^ Providing second-dose vaccination at 4–6 years of age led to further declines in varicella incidence, hospitalizations, and outbreaks in the US.^24^ It should be noted that, however, more than half of the 29 countries or areas with funded varicella vaccination adopt a one-dose schedule and most of those with two-dose vaccination have a second dose scheduled at ≥4 years [Information summarized from WHO, European Center for Disease Control, MSD and GSK].

In Hong Kong, the implementation of UVV has rapidly increased the first dose varicella vaccination uptake to about 99% for eligible preschool children.^10^ Reduction in varicella notification was observed shortly after universal vaccination, as the annual notification rate per 100,000 among children aged 3–5 had decreased from 1670 to 2916 between 2011 and 2013 and to 934–1256 between 2015 and 2018 (Supplementary Figure 3). Preschool outbreaks also decreased to a lower level but persisted in recent years (292–442 outbreaks annually from 2011 to 2013 compared with 111–284 outbreaks annually from 2015 to 2018). The Scientific Committee on Vaccine Preventable Diseases, the advisory body on immunization in Hong Kong, recently recommended advancing second-dose vaccines against measles, mumps, rubella, and varicella (given as combined MMRV) from Primary One to 18 months of age.^25^ Given the higher effectiveness of second-dose vaccination, this change in the program should reduce breakthrough infection and accumulation of susceptible children who experienced primary vaccine failure, and it would further limit varicella transmission and outbreaks in preschools. In addition, herpes zoster appeared to be less common among children vaccinated against varicella.^26,27^ Therefore, advancing the second-dose vaccine to 18 months of age should also bring a long-term impact of decreasing the probability of vaccinated children developing zoster later in their life.

We previously reported the burden of varicella having shifted to older children before UVV.^2^ The epidemiology of varicella and zoster is expected to change further as high varicella vaccination uptake has been achieved for children eligible for UVV. To assess the potential long-term impact of UVV on varicella and zoster, as well as different options to maximize the benefits of the vaccination program, we will model the transmission of VZV mechanistically. These local VE estimates provided important baseline data to monitor the impact of the UVV and could serve as important inputs for the mathematical model.

There were some limitations to this study. First, most varicella cases were ascertained clinically without laboratory confirmation. Breakthrough varicella with modified (less severe) symptoms is more difficult to be diagnosed clinically and is expected to increase with higher vaccination uptake. Inclusion of non-varicella cases will underestimate the VE. Second, varicella is generally a nonsevere disease and underreporting exists. We previously estimated that less than 50% of varicella infections were notified among those aged 3–5 years and the reporting sensitivity varied with time and age.^2^ This may bias our VE in different directions, depending on whether vaccinated cases are more likely to be reported. For instance, those who chose to actively vaccinate might be more aware of varicella and hence more likely to seek medical consultation and be reported. This would bias our VE estimates toward null. On the other hand, breakthrough varicella cases with milder symptoms may be less likely to seek medical consultation or require hospital admission. This will underestimate PCV and overestimate the VE. To our knowledge, we are not aware of reports on how varicella vaccination affects health-seeking and reporting behavior, though severe varicella in vaccinated children are rare.^28^ Third, complication and hospitalization alone might not be representative of clinical severity, as we did not collect further details such as the number of vesicles developed and the duration of hospitalization. Young children with varicella might be hospitalized for fever work-up instead of clinical severity. This would lead to underestimation of VE against admissions. Our VE estimates against complications were higher than that against hospital admissions. Varicella vaccine was shown to be more effective against severe outcomes, but severe disease in some observational studies was defined as varicella with ≥500 lesions or presence of complications/hospitalization.^6,22^ It should be noted that the median interval between interview and disease onset was only 3 days (interquartile range: 2–8 days) for cases included in our study. Cases that developed complications or hospitalized after data collection would not be counted as severe cases. Fourth, although adequate immunity against clinical disease might not have developed until 1 month after vaccination,^3^ cases that developed disease shortly after vaccination would still be regarded as vaccinated as the exact date of vaccination was not available for most varicella notifications. This will overestimate the PCV and underestimate the VE. Fifth, information on the exact vaccine product received by varicella cases was not collected and we were unable to estimate VE specific to different formulations. As children received varicella vaccines from different manufacturers (Table 1), our VE estimates were the effect of mixed vaccine products. Nevertheless, with the exception of varicella vaccine manufactured in South Korea, varicella vaccines available in Hong Kong are based on the Oka strain and their effectiveness are generally comparable.^6^ Sixth, ascertainment of vaccination status was different for the survey and notification. Vaccination status for surveyed children was ascertained by reviewing medical records, while that for most varicella notification was ascertained by parental recall. Thus, vaccination status of varicella cases was more prone to recall bias and it might affect the accuracy of PCV.

## Conclusion

We showed that varicella vaccine is effective in preventing varicella infection, complication, and hospitalization in Hong Kong, especially for two-dose vaccination. Countries with UVV should consider adopting a two-dose strategy with a short interval between the first and second doses to reduce breakthrough varicella and outbreaks in preschool. In view of the high VE, the epidemiology of varicella and herpes zoster is expected to change as universal vaccination program successfully rolls out in Hong Kong.
