# Supplementary material for: Varicella vaccine dose depended effectiveness and waning among preschool children in Hong Kong
Source: Hum Vaccin Immunother. 2019 Oct 23;16(3):499–505. doi: 10.1080/21645515.2019.1663121 (PMC7227687; doi:10.1080/21645515.2019.1663121)
Supplement: Supplemental Material [file khvi-16-03-1663121-s001.zip › KHVI_1663121_supplemental.pdf]

# Varicella Vaccine Dose Depended Effectiveness and Waning Among Preschool Children in Hong Kong

## Supplementary materials

**Supplementary Figure 1.** Imputation and data analysis for estimating varicella vaccine effectiveness in Hong Kong using the screening method

**Supplementary Figure 2.** Varicella vaccine effectiveness among preschool children aged 3 to 5 years in Hong Kong with different combinations on imputations and iterations

**Supplementary Figure 3.** Varicella vaccination uptake and varicella notification rate in Hong Kong. (a) First dose varicella vaccination uptake for preschool children aged three to five years in Hong Kong (except for 2001 when children included were aged four to five years). Annual varicella notification rate in Hong Kong from 1999 to 2018 for (b) children aged three to five years and (c) all ages.

**Supplementary Figure 1. Imputation and data analysis for estimating varicella vaccine effectiveness in Hong Kong using the screening method**

The main purpose of the imputation is to obtain the age of (hypothetical) vaccination for unvaccinated cases from the observed vaccination timing in the population i.e. survey respondents vaccinated against varicella. In order to obtain a dataset with the age of (hypothetical) vaccination for unvaccinated cases completely imputed, we first imputed missing values of vaccination-related variables among vaccination survey respondents and varicella cases to obtain complete datasets.

*Imputation of survey dataset (multiple imputation 1)*

Vaccination status was complete for all survey respondents. Among vaccinated survey respondents, less than 5% had no information on the received number of doses, the time of receiving first dose and the time of receiving their second dose. For imputation of survey data, dependent variables including vaccination status for second dose and age of vaccination for first and second dose was imputed based on predictor variables including survey year, age, type of preschool attended, place of birth, usual place of residence before 2 years of age and usual place of residence after 2 years of age (for 2015 survey only).

*Imputation of varicella notification dataset (multiple imputation 2)*

Across the three years of notification data in 2009, 2012 and 2015, between 6 and 15% of cases reported had missing information on either one of the vaccination-related variables (varicella vaccination status, number of doses (excluding year 2009 as this information was not collected) and age of last vaccination). We imputed missing values in varicella vaccination status, number of dose, and age of last varicella vaccination in the notification dataset, based on predictor variables including notification year and age (predictors used in imputing survey database such as type of preschool attended, place of birth, usual place of residence before 2 years of age and usual place of residence at time of survey was not available in the notification database).

*Imputation of age of (hypothetical) vaccination for unvaccinated cases (multiple imputation 3)*

After completing missing values of the survey and notification databases, we imputed the age of (hypothetical) vaccination for unvaccinated cases from vaccinated survey respondents using year and age as predictors.

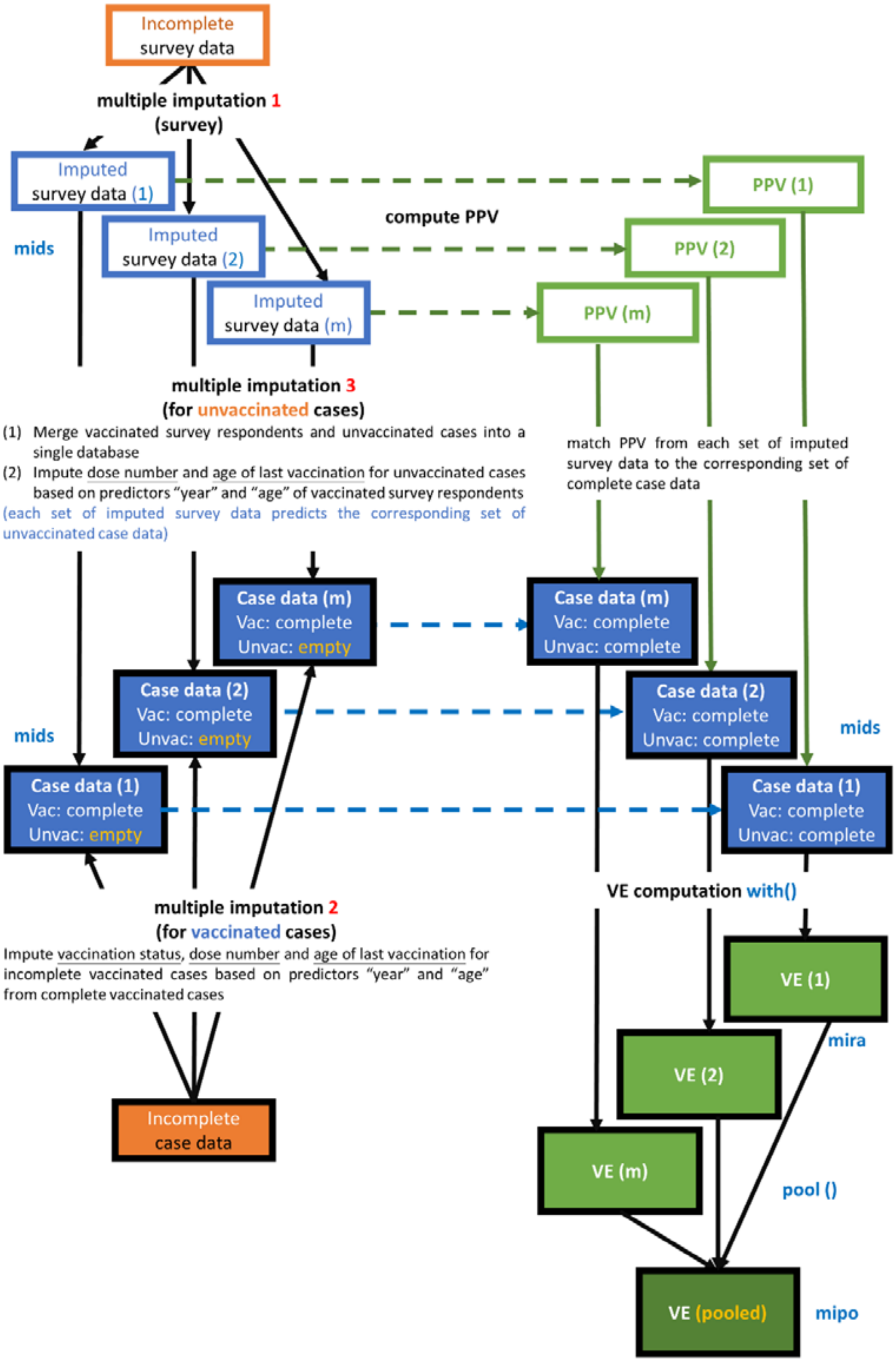

**Note:**

- All imputations was carried out using the mice (Multivariate Imputation by Chained Equations) v3.3.0 package from R. with() and pool() corresponds to the R syntax.
- Imputations 1 and 2 aimed at completing missing values related to vaccination variables in the survey and notification datasets.
- Imputation 3 aimed at imputing dose and age of vaccination for unvaccinated cases from notification dataset, with vaccinated respondents from the survey dataset as predictors.
- There was multiple number of imputations (m), which equaled to 500 (main result), 250 and 25 (as validity testing, please refer to Supplementary Figure 2). For simplicity only 3 boxes are drawn in the above figure.

**Abbreviations:**

PPV: proportion of population vaccinated

mids: Multiply Imputed Dataset

mira: Multiply Imputed Repeated Analyses

mipo: Multiple Imputation Pooled Object

**Supplementary Figure 2. Varicella vaccine effectiveness among preschool children aged 3 to 5 years in Hong Kong with different combinations on imputations and iterations**

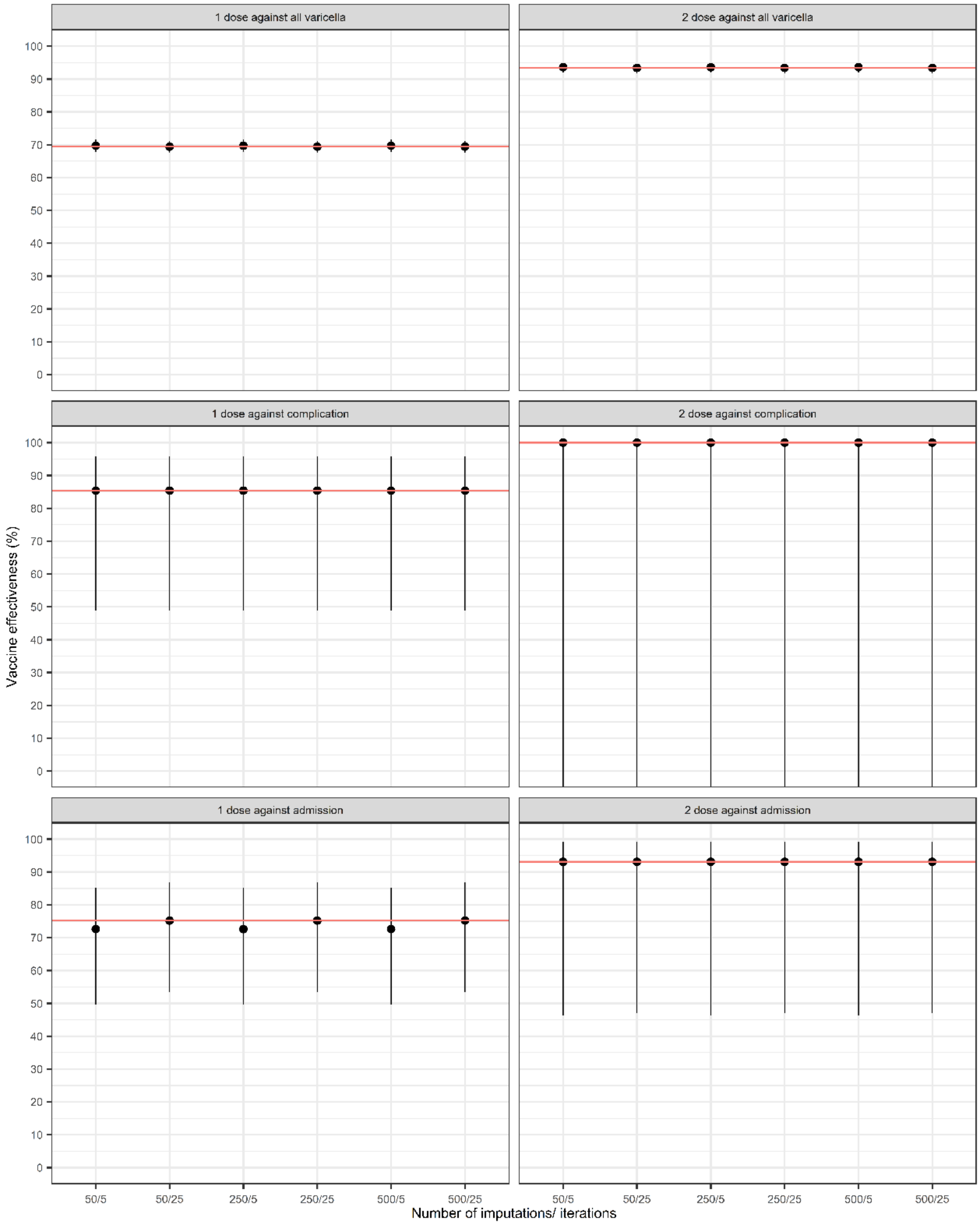


*Note: reference lines are the point estimate of the VE presented as main result in the manuscript (i.e. 500 imputations with 25 iterations in each imputation). Confidence intervals for "2 dose against complication" were –Inf to 100 for all combination of imputations and iterations.*

**Supplementary Figure 3. Varicella vaccination uptake and varicella notification rate in Hong Kong. (a) First dose varicella vaccination uptake for preschool children aged three to five years in Hong Kong (except for 2001 when children included were aged four to five years). Annual varicella notification rate in Hong Kong from 1999 to 2018 for (b) children aged three to five years and (c) all ages.**

(a) Varicella vaccination uptake among preschool children aged 3 to 5 years

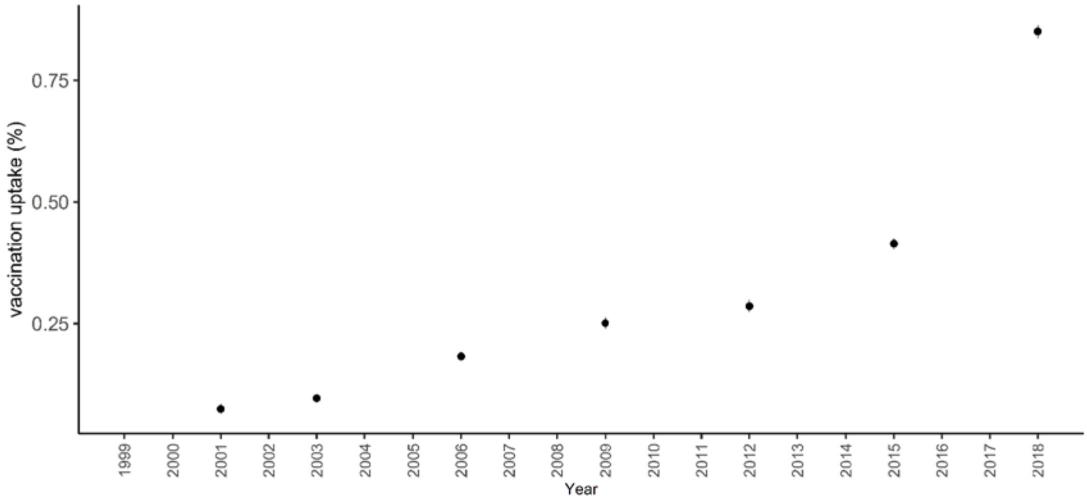


(b) 3 to 5 years

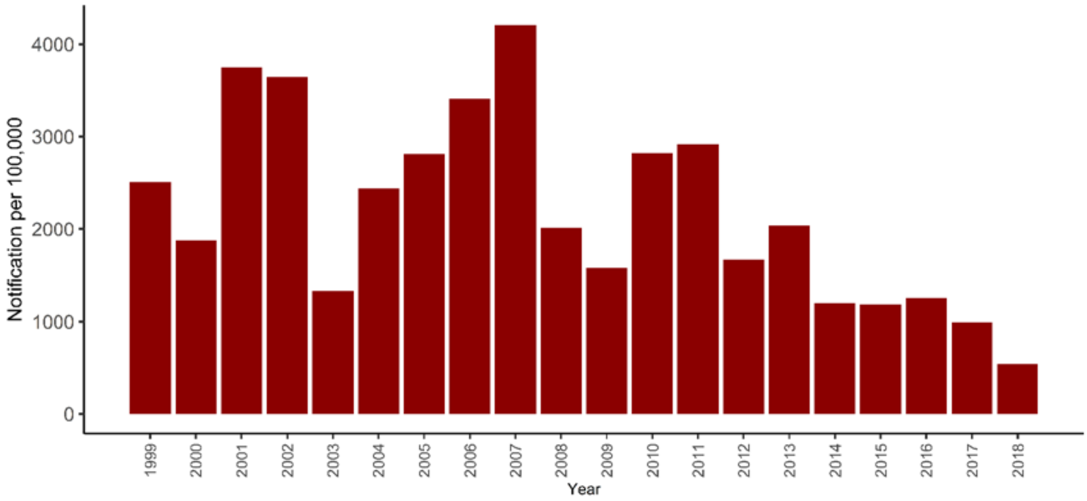

(c) All age

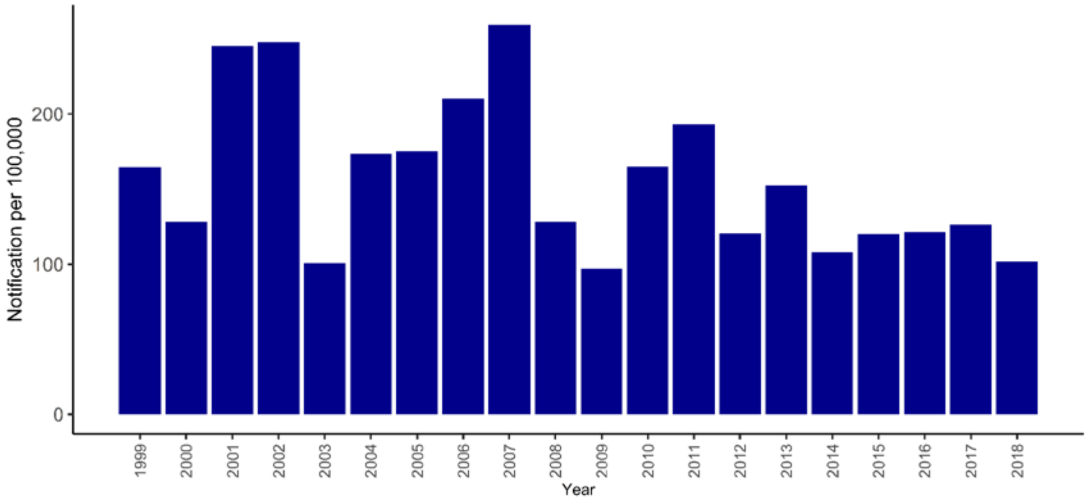


*Note: varicella notification for 1999 started in February.*
